# Translation and validation of the EORTC QLQ-BR45 among Ethiopian breast cancer patients

**DOI:** 10.1038/s41598-021-02511-9

**Published:** 2022-07-29

**Authors:** Mikiyas Amare Getu, Panpan Wang, Eva Johanna Kantelhardt, Edom Seife, Changying Chen, Adamu Addissie

**Affiliations:** 1 The First Affiliated Hospital, Zhengzhou University, Zhengzhou, 450052 Henan China; 2 School of Nursing and Health, Zhengzhou University, Zhengzhou, 450001 Henan China; 3 Institute of Medical Epidemiology, Biostatistics and Informatics, Martin-Luther-University, Halle (Saale), Germany; 4 Department of Gynecology, Martin-Luther-University, Halle (Saale), Germany; 5 Department of Oncology, Tikur Anbessa Specialized Hospital, College of Health Science, Addis Ababa University, Addis Ababa, Ethiopia; 6 Department of Preventive Medicine, School of Public Health, College of Health Sciences, Addis Ababa University, Addis Ababa, Ethiopia

**Keywords:** Cancer, Health care, Oncology

## Abstract

This study aimed to examine the validity and reliability of the EORTC QLQ-BR45 questionnaire among breast cancer patients in Ethiopia. This study included 248 breast cancer patients who completed the QLQ-BR45 and QLQ-C30 questionnaires. The internal reliability, test–retest reliability, and the content, concurrent, convergent, divergent, and clinical validity of the tool were examined. The statistical analyses included Cronbach’s α coefficient, Pearson’s correlation coefficient, standardised root mean square residual (SRMR), comparative fit index (CFI), t-test, and root mean square error of approximation (RMSEA). All items were marked as relevant, and item-level content validity index (I-CVI) scores ranged from 0.83 to 1. The S-CVI/Ave was calculated by dividing the sum of I-CVI values by the total number of items, which was found to be 0.94. The average CVR value was 0.76. The Cronbach’s α coefficient was 0.80 for all domains. All subscales met the minimal standards of reliability except the arm symptom scale (0.66). The test–retest reliability coefficient was 0.77 for all domains. Seven out of the 12 hypothesised scales showed positive correlations (r > 0.40) between the QLQ-BR45 and QLQ-C30 scales. Multitrait scaling analysis showed that the item-scale correlations exceeded the 0.40 criterion for item-convergent validity for 11 of the 12 hypothesised scales. The correlation coefficients between an item and its own subscale were significantly higher than with other subscales. The EORTC QLQ-BR45 had good reliability and validity, and it can be used to measure the quality of life of breast cancer patients in Ethiopia.

## Introduction

Cancer is a major cause of death worldwide, accounting for an estimated 18.1 million new cases and 9.6 million deaths in 2018^1^. Breast cancer is the most commonly diagnosed cancer and the leading cause of cancer deaths among women worldwide^2^. According to a report by the World Health Organization (WHO), breast cancer is the leading type of cancer in Ethiopia, with an estimated 15,244 (22.6%) new cases and a 5-year prevalence of 46.7 per 100,000^2^. Furthermore, the incidence of breast cancer increased between 1997 and 2012^3^.

Quality of life is a complex concept consisting of the individual’s physical health, psychological health, personal beliefs, social interactions, and relationship to their environment^4^. Measuring the quality of life of cancer patients is important for clinicians to predict the treatment response and survival time and to identify common problems. Better quality of life is associated with patient survival; thus, it could be considered a prognostic factor. Furthermore, the WHO suggests that quality of life should be considered as an endpoint in clinical trials of cancer patients. Recent studies have shown that the quality of life of breast cancer patients in Ethiopia is poor^5,6^.

EORTC QLQ-BR23 was one of the first modules developed for use in conjunction with the core questionnaire, the EORTC QLQ-C30^7^. It consists of 23 items and has been translated into more than 60 languages. However, knowledge about breast cancer has increased significantly since 1996, with major advances in its diagnosis and treatment; thus, the EORTC-BR23 needed to be updated^8^. Therefore, the EORTC Quality of Life Group (QLG) decided to update the BR23. The updated version of the original breast cancer module (QLQ-BR23), the EORTC QLQ-BR45, includes an additional 22 items. The final version of the EORTC QLQ-BR45 is currently available for use in clinical trials and practice, and it has been translated into 19 different languages^9^.

The original QLQ-BR23 was validated in Ethiopia 21 years after its original development^10^. Since its original development, there have been many advances in the diagnosis and treatment of breast cancer. In addition, the validation study of the original module in Ethiopia lacked test–retest reliability and criterion validity analyses. Therefore, this study aimed to translate, validate, and assess the psychometric properties of the EORTC QLQ-BR45 among breast cancer patients in Ethiopia.

## Methods

### Study design

Institutional based longitudinal study was conducted.

### Study setting

The study took place in oncology centres in Ethiopia.

### Inclusion and exclusion criteria

Histologically confirmed female breast cancer patients aged 18 years and above who were receiving or had previously received curative or palliative treatment, who had no previous primary or recurrent tumour, and who could understand and speak the Amharic language were invited to participate in the study. Patients were excluded from the study if they had a history of mental illness or cognitive impairment, if they were not willing to participate, or if they had any other severe medical illnesses, coexisting malignancies, or other metastatic disease.

### Sample

The minimum sample size recommendations for validation studies range from 100 to 400 participants or more^11,12^. According to the EORTC, the sample size is determined by the number of items in the questionnaire. The sample was calculated according to the EORTC guidelines and the recommendations for multivariate psychometric analysis, which concluded that the sample size needed to be five to ten times the number of items^13^. Therefore, the calculated sample size was 248.

### Instruments

#### EORTC QLQ-C30

The EORTC QLQ-C30 is a core questionnaire that assesses the quality of life of cancer patients. It consists of a 30-item questionnaire composed of five functional scales, three symptom scales, and a global health and quality-of-life scale. The other single item symptoms include dyspnea, loss of appetite, sleep disturbance, constipation, diarrhoea, and financial difficulties. The questionnaire was translated into Amharic, the official language of Ethiopia, and validated to assess the quality of life of Ethiopian cancer patients^14^.

#### EORTC QLQ-BR45

EORTC QLQ-BR45 is a specific breast cancer module that is used in combination with the EORTC QLQ-C30 core questionnaire. The EORTC QLG updated the previous breast cancer-specific module to EORTC QLQ-BR45. The updated version incorporates an additional 22 items, including a target symptom scale and a satisfaction scale. These new items include two multi-item scales: target symptom scale (20 items) and satisfaction scale (two items). The target symptom scale can be further divided into three subscales: endocrine therapy scale, endocrine sexual scale, and skin/mucosa scale^9^. A formal permission letter was obtained from the authors^9,15^.

The item scoring procedure for the EORTC QLQ-C30 and the EORTC QLQ-BR45 was managed according to the EORTC QLQ-C30 scoring manual. After the scoring procedures, the score was transformed into a 0–100 scale. A high score for functional scales indicates a high level of functioning, while for symptom scales, a higher score indicates a higher level of symptoms^16^.

### Translation procedure

The translation procedure for the EORTC QLQ BR-45 was based on the EORTC QLG translation procedure^17,18^. The English to Amharic translators (forward translators) were given the original English version. The original English version of the EORTC QLQ BR-45 was translated into Amharic by two oncologists independently of one another. The forward translation was performed by two separate translators who independently translated the questionnaire from English into Amharic. The two translations were reconciled by the principal investigator and then translated back into English by another oncology physician and a nurse, independently. The two translators were given the reconciled translation and were blinded to the original English version. The preliminary translation was reviewed by a professional proofreader. The proofreader checked the equivalence between the original English version questionnaire and the preliminary translation. The interim analysis was prepared after the translation unit members, the proofreader, and the principal investigator reached an agreement on the preliminary translation.

### Pilot testing

The translated questionnaire was pilot tested on 10 female breast cancer patients. The principal investigator discussed with the participants whether the translation was difficult to understand, difficult to answer, upsetting/offensive, or confusing. The comments suggested by the participants were back-translated into English. After the pilot testing was successfully completed, the translation unit sent the final translation to the principal investigator for approval and use.

### Ethical approval

Ethical approval was obtained before conducting the study. Participants’ information was kept confidential. There was no risk associated with participating in the study.

### Statistical analysis

The Amharic version of QLQ-BR45 questionnaire was evaluated for its internal reliability, test–retest reliability, content validity, concurrent validity, convergent validity, divergent validity, and known-group validity.

Content validity was evaluated by a panel of six experts from September to November 2020^19^, including a professor of public health, an oncology nurse, an assistant professor in clinical pharmacology, and a PhD candidate in pharmacology with experience in the validation of QoL instruments. These experts were chosen based on their clinical and research experience.

The content validity index was evaluated at two levels, namely item-level content validity index (I-CVI) and scale-level content validity index (S-CVI), based on expert review. The S-CVI has two extensions: universal agreement (S-CVI/UA) and average (S-CVI/Ave). Use of the S-CVI/Ave is recommended, and acceptable values of S-CVI/Ave are 0.90 or higher^20^. An I-CVI value of 0.78 or higher is considered excellent^21,22^.

The content validity ratio (CVR) ranges from −1 to 1. Higher scores indicate greater agreement of panellists on the necessity of an item in an instrument. The closer the CVR is to 1, the more essential the tool will be. The formula for the CVR is CVR = (Ne–N/2)/(N/2), where Ne is the number of panel members considered “essential” and N is the total number of panellists. The numeric value of the CVR was determined using the Lawshe table^23^.

Concurrent validity means the agreement with the true value. The new questionnaire was compared to well-established instruments that already have an estimated validity. The concurrent validity is considered to be high if the agreement or correlation between the EORTC QLQ-BR45 and EORTC QLQ-C30 is high^12^.

Convergent validity is defined as a Pearson correlation coefficient between the item and its own scale (item-scale correlation) higher than r ≥ 0.40, while divergent validity is indicated when the relationship of one item to its domain is significantly higher than its relationship to another scale^15^.

Confirmatory factor analysis was used to test whether the correlation corresponds to the hypothesised scale structure. This method tests whether the hypothesised relationship between observed variables and their underlying latent dimensions is confirmed. The comparative fit index (CFI) is equal to the discrepancy function adjusted for sample size. The CFI ranges from 0 to 1, with larger values indicating a better model fit. An acceptable model fit is indicated by CFI values of 0.90 or greater. The root mean square error of approximation (RMSEA) is related to the residual in the model. RMSEA values range from 0 to 1, with an RMSEA value of 0.06 or less considered an acceptable model fit^24^.

Known-group comparisons were performed to evaluate how well scales can discriminate between participants enrolled in different groups, according to their age, residence, disease stage, and treatment modalities^25^. This psychometric property is also called sensitivity.

The internal consistency of the multi-item scale was assessed by Cronbach’s α coefficient^26^. As recommended, a Cronbach’s α coefficient of 0.70 or greater is acceptable^12,26^, while values exceeding 0.80 are considered good^27^. A subgroup of follow-up patients with no change in health status (stable health status) was invited to complete the QLQ-C30 and QLQ-BR45 a second time one to two weeks later for the test–retest analysis. This analysis was used to test the consistency of the module based on a repeatable score at a different time^27^. Thirty patients participated in the test–retest analysis one to two weeks after the first assessment.

### Ethics approval

All procedures performed in studies involving human participants were in accordance with the ethical standards of the institutional and/or national research committee and with the 1964 Helsinki declaration and its later amendments or comparable ethical standards. The study was approved by Zhengzhou University IRB (number: ZZURIB 2020-10; Date: 18/06/2020) and Addis Ababa University, College of Health Science teaching hospital (number: 101/20/Onco; Date: 28/10/2020).

### Consent to participate

Informed consent was obtained from all individual participants included in the study.

### Consent to publication

The authors affirm that human research participants provided informed consent for publication.

## Result

### Sociodemographic and clinical characteristics

Out of 248 breasts cancer patients, 240 patients agreed to participate in the study, with a total response rate of 96.8%. The age of the participants ranged from 23 to 85 years, with a mean age of 44.7 years (SD 11.2 years).

Regarding the educational level of study participants, 75 (31.4%) had no education, 51 (21.3%) had primary school education, 57 (23.8%) had secondary school education, and 56 (23.4%) had above secondary school education. Of the 240 participants, 139 (57.9%) were married. Most of the participants (59%) were followers of the Orthodox Christianity faith. The majority of participants (76.6%) resided in an urban area.

Two hundred and eleven (95.5%) participants had undergone or were undergoing chemotherapy. Most of the study participants (40.7%) had a stage III tumour. Of the participants, 201 (83.8%) had no other illnesses or comorbidities, and 96.7% of participants were under treatment follow-up (Table 1).

**Table 1:** Socio-demographic and clinical characteristics of breast cancer patients in Ethiopia, 2021 (N = 240).

Variable	Frequency	Percentage	
**Age**			
Mean (SD)	*44.7 (11.2)*		
Range	23–85		
**Educational status**			
No education	75	31.2	
Primary school education	51	21.2	
Secondary school education	57	23.8	
Above secondary school education	57	23.8	
**Religion**			
Orthodox christianity	142	59.2	
Protestant	50	20.8	
Muslim	41	17.1	
Catholic	4	1.7	
Other^†^	3	1.2	
**Occupational status**			
Housewife	135	56.3	
Government employed	35	14.6	
Farmer	25	10.4	
Merchant	15	6.3	
Daily laborer	13	5.4	
Other^‡^	17	7.1	
**Residence**			
Urban	184	76.7	
Rural	56	23.3	
**Marital status**			
Single	21	8.8	
Married	139	57.9	
Divorced	34	14.2	
Widowed	46	19.2	

Clinical characteristics			
Treatment*			
Surgery	201	33.4	
Radiotherapy	72	12.0	
Chemotherapy	228	37.9	
Hormonal therapy	101	16.8	
**Stage of tumor **			
Stage I	23	9.6	
Stage II	87	36.3	
Stage III	98	40.8	
Stage IV	32	13.3	
**Admission status **			
New	8	3.3	
Follow-up	232	96.7	
**Comorbidity **			
Yes	39	16.2	
No	201	83.8	
Total	240	100	

^†^Jehovah’s witnesses.

^‡^Private organization.

*The sum of frequency is greater than total because it is multiple response items.

### Reliability

The mean and standard deviation of each subscale/item, Cronbach’s α coefficient, and test–retest reliability coefficients intraclass correlation (ICC) and correlation coefficient r) of all domains are presented in Table 2. The Cronbach’s α coefficient of the Amharic version of the EORTC QLQ-BR45 was 0.80. All of the domains had an acceptable internal consistency value greater than 0.7, except for arm symptoms (0.66). The test–retest reliability coefficient was 0.768 for all domains. The test–retest reliability coefficients of most domains were less than 0.70, except for BRST (0.73), BRBS (0.75), and BRET (0.72). The ICC was similar to the correlation coefficient r, indicating no significant drift in the mean response for all domains.

**Table 2:** Reliability of EORTC QLQ BR45 Amharic version among breast cancer patients in Ethiopia, 2021.

Domain (subscales/item)	Item/s no	Mean (± SD)	Internal consistency (Cornabach’s α coefficient)	Test–retest reliability (correlation coefficient)	Intra-class correlation (95%CI)
**Functioning scales**					
Body image (BRBI)	9–12	78.6 (25.9)	0.90	0.68	0.68 (0.44–0.83)
Sexual functioning (BRSEF)	14, 15	85.4 (21.9)	0.76	0.65	0.64 (0.37–0.81)
Sexual enjoyment (BRSEE)^†^	16	85.5 (26.3)	–	0.65	0.62 (0.35–0.80)
Future perspective (BRFU)	13	57.1 (37.3)	–	0.35	0.35 (−0.01 to 0.62)
**Symptoms scales/item**					
Systemic therapy side effects (BRST)	1–4, 6, 7, 8	29.0 (20.0)	0.74	0.73	0.73 (0.50–0.86)
Breast symptoms (BRBS)	20–23	19.9 (25.3)	0.80	0.65	0.65 (0.39–0.82)
Arm symptoms (BRAS)	17,18,19	22.7 (23.1)	0.66	0.36	0.35 (0.01–0.62)
Upset by hair loss (BRHL)^‡^	5	41.8 (34.6)	–	0.54	0.55 (−0.09 to 0.87)
**Satisfaction scale**					
Breast satisfaction scale (BRBS)	44, 45	70.8 (29.0)	0.85	0.75	0.76 (0.55–0.88)
**Target symptom scale**					
Endocrine therapy scale (BRET)	24–26, 33–39	17.7 (15.9)	0.81	0.72	0.72 (0.49–0.86)
Endocrine sexual scale (BRES)	40–43	7.1 (14.7)	0.72	0.34	0.32 (−0.03 to 0.60)
Skin/mucosis scale (BRSM)	27–32	14.3 (15.5)	0.70	0.12	0.12 (−0.24 to 0.45)

^†^BRSEE, sexual enjoyment, is not applicable if item 15 is “not at all”.

^‡^BRHL, upset by hair loss, is not applicable if item 4 is “not at all”.

*CI* confidence interval.

### Content validity

#### Item-level content validity index

The I-CVI is computed as the number of experts giving a rating of 3 or 4 (quite or highly relevant) to the relevance of each item, divided by the total number of experts. The I-CVI for the relevance of each item was greater than 0.78^21,22^. All items were marked as relevant, and the I-CVI score ranged from 0.83 to 1. Thirty items had an I-CVI score of 1.00 and 15 had a score of 0.83.

#### Scale-level content validity index/average

The S-CVI/Ave was calculated by dividing the sum of I-CVI values by the total number of items, which was found to be 0.94.

#### Content validity ratio

The CVR was generated for each item. According to the Lawshe table^23^, the minimum CVR value for a total number of six panellists was 0.99. Sixteen items had a CVR value of 1.00, 27 items had a score of 0.67, and two items had a score of 0.33. The average CVR value was 0.76.

#### Clarity

Clarity was assessed by the six panelists on a 3-point Likert scale (1, not clear; 2, somewhat clear; 3, very clear). The average clarity scores for individual items ranged from 2.5 to 3, with 32 (71.1%) items considered very clear. Overall, 32 items had an average clarity score of 3.00, six items had a score of 2.83, four items had a score of 2.67 and two had a score of 2.5.

### Construct validity

#### Multitrait scaling analysis

##### Item scale correlation

Table 3 shows the item-scale correlation of the EORTC QLQ-BR45. Item-scale correlations (corrected for overlap) exceeded the 0.40 criterion for item-convergent validity for 11 of the 12 hypothesised scales, with the exception of item 38.

**Table 3:** Item-scale correlation of EORTC QLQ BR45 Amharic version among breast cancer patients in Ethiopia, 2021.

Item number	Description	BRST	BRBS	BRAS	BRHL	BRBI	BRSEF	BRSEE	BRFU	BRET	BRES	BRSM	BRBSt
1	Dry mouth	0.652**	0.187**	0.212**	0.208*	0.286**	−0.022	−0.097	0.130*	0.314**	0.204**	0.286**	−0.030
2	Food and drink test	0.769**	0.344**	0.282**	0.365**	0.261**	0.029	−0.004	0.198**	0.462**	0.302**	0.363**	−0.005
3	Eyes painful, irritated or watery	0.638**	0.256**	0.247**	0.107	0.207**	−0.044	−0.045	0.122	0.423**	0.154*	0.335**	−0.024
4	Loss of hair	0.629**	0.250**	0.168**	0.268**	0.292**	0.008	−0.056	0.118	0.319**	0.131*	0.315**	−0.106
6	Felt ill/unwell	0.639**	0.381**	0.441**	0.430**	0.508**	−0.054	−0.054	0.412**	0.541**	0.200**	0.421**	−0.047
7	Hot flushes	0.588**	0.254**	0.232**	0.435**	0.373**	−0.069	−0.032	0.354**	0.424**	0.204**	0.223**	0.067
8	Headache	0.574**	0.289**	0.211**	0.211*	0.214**	−0.012	0.005	0.158*	0.283**	0.185**	0.224**	−0.047
20	Pain in affected breast	0.425**	0.846**	0.529**	0.298**	0.377**	−0.064	−0.066	0.391**	0.414**	0.138*	0.271**	−0.019
21	Breast swollen	0.293**	0.851**	0.372**	0.234**	0.266**	−0.059	−0.043	0.230**	0.362**	0.102	0.205**	−0.057
22	Breast over sensitive	0.378**	0.849**	0.422**	0.284**	0.358**	−0.066	−0.055	0.262**	0.374**	0.084	0.264**	0.061
23	Skin problem	0.342**	0.501**	0.323**	0.323**	0.309**	0.027	0.001	0.128*	0.311**	0.204**	0.302**	−0.017
17	Pain in arm/shoulder	0.401**	0.445**	0.813**	0.233**	0.242**	−0.161*	−0.111	0.276**	0.433**	0.148*	0.372**	0.088
18	Swollen arm/hand	0.122	0.283**	0.690**	0.160	0.239**	0.049	0.139*	0.156*	0.284**	0.167**	0.334**	−0.029
19	Problem in raising arm	0.337**	0.469**	0.815**	0.195*	0.201**	−0.050	0.035	0.209**	0.384**	0.108	0.362**	−0.025
5	Upset by hair-loss	0.461**	0.324**	0.249**	1.000**	0.517**	0.026	−0.014	0.419**	0.373**	0.116	0.267**	−0.061
9	Physically less attractive	0.497**	0.334**	0.294**	0.517**	0.850**	0.019	−0.041	0.497**	0.320**	0.218**	0.358**	−0.141*
10	less feminine	0.385**	0.348**	0.235**	0.436**	0.858**	0.086	0.005	0.491**	0.371**	0.233**	0.309**	−0.163*
11	Problem looking self	0.348**	0.348**	0.240**	0.378**	0.907**	0.154*	0.034	0.551**	0.372**	0.146*	0.311**	−0.152*
12	Dissatisfied with your body	0.424**	0.350**	0.259**	0.477**	0.887**	0.145*	0.004	0.594**	0.334**	0.196**	0.293**	−0.148*
14	Interested in sex	0.009	−0.004	−0.096	0.008	0.182**	0.895**	0.605**	0.078	−0.034	0.117	0.039	0.007
15	Sexually active	−0.065	−0.127*	−0.052	0.039	0.028	0.900**	0.713**	0.038	−0.088	0.156*	0.031	−0.021
16	Sexual enjoyment	−0.064	−0.065	0.012	−0.014	0.001	0.735**	1.000**	0.010	−0.001	0.074	0.020	−0.013
13	Future perspective	0.318**	0.349**	0.282**	0.419**	0.610**	0.064	0.010	1.000**	0.351**	0.099	0.221**	−0.032
24	Sweating	0.316**	0.355**	0.265**	0.166	0.186**	−0.083	−0.090	0.151*	0.494**	0.099	0.287**	−0.010
25	Mood swings	0.416**	0.433**	0.214**	0.363**	0.352	0.000	−0.084	0.312**	0.567**	0.171**	0.271	0.042
26	Dizziness	0.414**	0.378**	0.228**	0.202*	0.268	−0.061	−0.069	0.173**	0.533**	0.083	0.339**	−0.101
33	Problem with joint	0.475**	0.096	0.301**	0.301**	0.267	−0.035	−0.038	0.228**	0.683**	0.194**	0.587**	0.043
34	Stiffness with joint	0.434**	0.212**	0.371**	0.168	0.197	−0.083	0.006	0.095	0.708**	0.134*	0.564**	−0.023
35	Joint pain	0.445**	0.224**	0.377**	0.198*	0.276	−0.044	0.060	0.239**	0.769**	0.161*	0.553**	0.016
36	Bone ache	0.454**	0.377**	0.463**	0.256**	0.344	−0.073	0.015	0.276**	0.765**	0.162*	0.652**	0.027
37	Muscle ache	0.480**	0.355**	0.487**	0.234**	0.309	−0.103	−0.016	0.313**	0.726**	0.258**	0.601**	−0.039
38	Weight gain	0.047	0.166**	0.046	0.117	0.082	0.087	0.115	0.174**	0.372**	0.017	0.055	0.017
39	Weight gain been a problem	0.149*	0.108	0.121	0.226**	0.099	−0.012	0.128	0.142*	0.405**	0.070	0.102	−0.027
40	Dry vagina	0.206**	0.083	0.118	0.012	0.161*	0.154*	0.053	0.023	0.158*	0.843**	0.134*	−0.114
41	Discomfort in vagina	0.299**	0.144*	0.189**	0.158	0.226**	0.151*	0.100	0.105	0.271**	0.874**	0.240**	−0.018
42	Pain during sex	0.160	0.246*	−0.072	0.049	0.142	−0.156	−0.286*	0.080	0.069	0.562**	0.026	−0.134
43	Dry vagina during sex	0.273**	0.068	−0.025	−0.167	0.053	−0.044	−0.113	−0.004	0.203	0.791**	0.224*	−0.101
27	Soreness in mouth	0.247**	0.051	0.102	0.097	0.209**	0.027	−0.049	0.091	0.292**	0.008	0.578**	−0.118
28	Redness in mouth	0.216**	0.095	0.152*	0.076	0.130*	0.033	0.107	0.026	0.287**	0.042	0.464**	−0.100
29	Pain in hands/feet	0.423**	0.320**	0.431**	0.288**	0.309**	0.065	0.018	0.210**	0.532**	0.265**	0.676**	0.077
30	Redness in hands/feet	0.257**	0.228**	0.275**	0.112	0.350**	0.032	0.011	0.246**	0.388**	0.167**	0.500**	−0.081
31	Tingling in fingers/toes	0.357**	0.185**	0.353**	0.194*	0.223**	−0.036	−0.048	0.115	0.463**	0.183**	0.774**	−0.151*
32	Numbness in fingers/toes	0.297**	0.168**	0.326**	0.212*	0.183**	0.033	0.046	0.138*	0.524**	0.103	0.770**	−0.115
44	Cosmetic result of surgery	0.025	−0.005	0.040	−0.004	−0.147*	−0.042	−0.029	−0.029	0.040	−0.055	−0.041	0.938**
45	Skin appearance	−0.082	0.004	0.018	−0.102	−0.182**	0.028	0.039	−0.044	−0.018	−0.061	−0.144	0.938**

Systemic therapy side effects (BRST); Breast symptoms (BRBS); Arm symptoms (BRAS); upset by hair loss (BRHL); Body image (BRBI); Sexual functioning (BRSEF); Sexual enjoyment (BRSEE); Future perspective (BRFU); Endocrine therapy scale(BRET); Endocrine sexual(BRES); Skin mucosis (BRSM); and Breast satisfaction(BRBSt).

Significant values are in bold.

The correlation coefficients between an item and its own subscale were significantly higher than for other subscales. Item convergence and discrimination were noted in 97.8% and 88.7% of QoL scales, respectively. The most obvious scaling failure corresponded to systematic therapy side effects. The small number of scaling errors provided strong support for the hypothesised scale structure of the EORTC QLQ-BR45.

#### Interscale correlations

Table 4 presents the correlations among the 12 scales of the QLQ-BR45. All correlation coefficients ranged from 0.001 to 0.735. A strong correlation coefficient (r = 0.735) was found between the sexual enjoyment and sexual functioning scales.

**Table 4:** Inter-scale correlation of EORTC QLQ BR45 Amharic version among breast cancer patients in Ethiopia, 2021.

Subscales	BRST	BRBS	BRAS	BRHL	BRBI	BRSEF	BRSEE	BRFU	BRET	BRES	BRSM	BRBSt
BRST												
BRBS	0.432**											
BRAS	0.384**	0.522**										
BRHL	0.461**	0.324**	0.249**									
BRBI	0.472**	0.394**	0.293**	0.517**								
BRSEF	**−0.032**	**−0.074**	**−0.082**	**0.026**	**0.116**							
BRSEE	−0.064	−0.065	0.012	−0.014	0.001	0.735**						
BRFU	0.318**	0.349**	0.282**	0.419**	0.610**	0.064	0.010					
BRET	0.606**	0.452**	0.480**	0.373**	0.399**	−0.068	−0.001	0.351**				
BRES	0.300**	0.128*	0.180**	0.116	0.226**	0.152*	0.074	0.099	0.225**			
BRSM	0.481**	0.291**	0.460**	0.267**	0.363**	0.039	0.020	0.221**	0.673**	0.221**		
BRBSt	−0.051	−0.006	0.022	−0.061	−0.172**	−0.008	−0.013	−0.032	−0.006	−0.081	−0.114	

Systemic therapy side effects (BRST); Breast symptoms (BRBS); Arm symptoms (BRAS); upset by hair loss (BRHL); Body image (BRBI); Sexual functioning (BRSEF); Sexual enjoyment (BRSEE); Future perspective (BRFU); Endocrine therapy scale(BRET); Endocrine sexual(BRES); Skin mucosis(BRSM); and Breast satisfaction(BRBSt).

Significant values are in bold.

The endocrine therapy scale and systematic therapy side effect scales were strongly correlated with most of the other subscales. The endocrine therapy scale had a strong correlation with systematic therapy side effects (r = 0.61), breast symptoms (r = 0.45), arm symptoms (r = 0.48), and body image (r = 0.40). The systematic therapy side effect scale was correlated with the breast symptom (r = 0.43), hair loss (r = 0.46), body image (r = 0.47), endocrine therapy (r = 0.61), and skin mucosis (r = 0.48) scales.

### Criterion validity

The Pearson’s correlation coefficients of scores between the domains of the two instruments (QLQ-BR45 and QLQ-C30) are presented in Table 5. Seven out of the 12 hypothesised scales showed correlations (r > 0.40) between the QLQ-BR45 and QLQ-C30 scales. The instrument correlations were higher between the same and similar domains than between different and non-similar domains. For example, the systematic therapy side effect scale was strongly correlated to the conceptually related QLQ-C30 fatigue, nausea and vomiting, and pain scales, with correlation coefficients greater than 0.5. The breast symptom and arm symptom scales also had strong correlations with pain (r > 0.5). All hypothesised correlations were statistically significant (p < 0.01). The QLQ-BR45 scales showed comparatively low correlations (r < 0.40) with QLQ-C30 scales in 144 out of 180 comparisons (80%). The QLQ-BR23 endocrine therapy scales showed strong correlations with the QLQ-C30 role functioning (r = 0.45), emotional functioning (r = 0.40), cognitive functioning (r = 0.44), fatigue (r = 0.53), and pain (r = 0.51) scales.

**Table 5:** Inter-scale correlations among scales of EORTC QLQ BR45 and with scales of EORTC QLQ-C30 Amharic version among breast cancer patients in Ethiopia, 2021.

QLQ BR45 domains	QLQ C-30 domains														
	PF	RF	EF	CF	SF	FA	NV	PA	DY	SL	AP	CO	DI	FI	QL
Systematic therapy side effects	0.302**	0.442**	0.584**	0.548**	0.359**	0.545**	0.514**	0.545**	0.262**	0.237**	0.422**	0.307**	0.298**	0.207**	−0.374**
Breast symptom s	0.194**	0.303**	0.368**	0.299**	0.145*	0.415**	0.235**	0.501**	0.118	0.069	0.092	0.096	0.071	0.269**	−0.170**
Arm symptoms	0.274**	0.362**	0.306**	0.343**	0.187**	0.441**	0.262**	0.522**	0.215**	0.162*	0.208**	0.103	0.064	0.220**	−0.185**
Upset by hair loss	0.028	0.230**	0.374**	0.302**	0.338**	0.366**	0.205*	0.348**	0.131	0.198*	0.078	0.178*	−0.020	0.197*	−0.262**
Body image	0.049	0.259**	0.531**	0.310**	0.328**	0.358**	0.218**	0.379**	0.075	0.115	0.194**	0.210**	0.008	0.293**	−0.313**
Sexual functioning	−0.242**	−0.111	−0.061	−0.115	−0.153*	−0.140*	−0.159*	−0.116	−0.121	−0.183**	−0.166**	−0.050	−0.110	−0.041	0.157*
Sexual enjoyment	−0.219**	−0.143*	−0.083	−0.061	−0.173**	−0.102	−0.181**	−0.070	−0.154*	−0.218**	−0.177**	−0.106	−0.047	−0.032	0.143*
Future perspective	0.025	0.241**	0.460**	0.238**	0.271**	0.285**	0.217**	0.290**	0.046	0.132*	0.149*	0.161*	−0.035	0.196**	−0.204**
Endocrine therapy symptoms	0.299**	0.443**	0.505**	0.438**	0.271**	0.532**	0.315**	0.509**	0.182**	0.153*	0.267**	0.184**	0.270**	0.208**	−0.334**
Endocrine sexual symptoms	0.049	0.185**	0.131*	0.101	0.145*	0.068	0.049	0.167**	0.070	0.079	0.002	0.148*	0.058	0.004	−0.075
Skin mucosis symptoms	0.348**	0.454**	0.404**	0.408**	0.249**	0.451**	0.381**	0.481**	0.232**	0.149*	0.361**	0.186**	0.153*	0.211**	−0.321**
Breast satisfaction	−0.005	−0.017	−0.082	0.005	−0.153*	−0.025	−0.093	0.025	−0.022	0.016	−0.059	0.033	0.066	−0.099	0.049

*PF* physical functioning, *RF* role functioning, *EF* emotional functioning, *CF* cognitive functioning, *SF* sexual functioning, *FA* fatigue, *NV* nausea and vomiting, *PA* pain, *DY* dyspnea, *SL* sleep lack, *AP* loss of appetite, *CO* constipation, *DI* diarrhea, *FI* fatigue, *QL* quality of life .

Significant values are in bold.

*P < 0.05, **P < 0.01.

### Clinical validity (known-group comparisons)

Independent samples t-test examined the statistical significance of group differences according to the age, residence, disease stage, and treatment modalities of patients. Sexual functioning (p = 0.02) and sexual enjoyment (p = 0.05) scores were significantly lower in patients with stage IV cancer than in those with stage I cancer. No other statistically significant differences were observed between cancer stages. Study participants over 45 years of age reported better sexual functioning (p < 0.001), sexual enjoyment (p < 0.001), body image (p = 0.003), and future perspectives (p = 0.009) and lower symptom scores (upset by hair loss, p = 0.031) than participants aged under 45 years. The endocrine sexual-related function (p = 0.031) was worse in patients residing in rural areas than those in urban areas.

### Confirmatory factor analysis

#### Model assumptions

To verify the stability and rationality of QLQ-BR45, the hypothesised scale structure was assumed to be a good model.

However, the hypothesised scale structure model fit was poor according to CFA model fit indicators (CFI = 0.75, RMSEA = 0.08, SRMR = 0.09). These results indicate that the hypothesised scale structure did not fit the model well.

#### Modification and model fit

According to the modification indices, seven covariance correlations were added to the model, and each covariance correlation was between the residuals of different items in the same dimension, which supports the hypothesised scale.

In this study, the confirmatory factor analysis showed that the structure of the QLQ-BR45 model with goodness of fit chi-square/df was 2.06; (P < 0.001), RMSEA was 0.05, CFI was 0.89, and standardised root mean square residual (SRMR) was 0.08, CMIN/DF 2.067. The values of these indicators demonstrate a good model fit. The estimated item factor loadings for the final model using the modification indices are reported in Table 6. The endocrine therapy subscale covaried significantly with skin mucosis (β = 0.175, p < 0.001), systematic therapy side effect (β = 0.131, p < 0.001), body image (β = 0.113, p < 0.001), arm symptom (β = 0.136, p < 0.001), and breast symptom (β = 0.118, p < 0.001) scales. The skin mucosis scale covaried significantly with systematic therapy side effect (β = 0.186, p < 0.001), body image (β = 0.192, p < 0.001), arm symptom (β = 0.216, p < 0.001), and breast symptom (β = 0.165, p < 0.001) scales. The endocrine sexual scale covaried significantly with sexual functioning (β = 0.248, p < 0.001).

**Table 6:** Standardized item factor loadings for EORTC QLQ BR45 model Amharic version among breast cancer patients in Ethiopia, 2021.

	Std. factor loading	P value
**Endocrine therapy scale**		
Item 39	0.321	< 0.001
Item 38	0.402	< 0.001
Item 37	0.451	< 0.001
Item 36	0.688	< 0.001
Item 35	0.747	< 0.001
Item 34	0.757	< 0.001
Item 33	0.818	< 0.001
Item 26	0.755	< 0.001
Item 25	0.100	< 0.001
Item 24	0.192	< 0.001
**Skin mucosis scale**		
Item 29	0.577	< 0.001
Item 30	0.466	< 0.001
Item 27	0.456	< 0.001
Item 32	0.738	< 0.001
Item 31	0.710	< 0.001
Item 28	0.375	< 0.001
**Endocrine sexual scale**		
Item 42	0.924	< 0.001
Item 43	0.944	< 0.001
Item 41	0.232	< 0.001
Item 40	0.251	< 0.001
**Breast satisfaction scale**		
Item 44	0.785	< 0.001
Item 45	0.726	< 0.001
**Systematic therapy side effects**		
Item 1	0.563	< 0.001
Item 2	0.719	< 0.001
Item 3	0.547	< 0.001
Item 4	0.561	< 0.001
Item 6	0.690	< 0.001
Item 7	0.513	< 0.001
Item 8	0.465	< 0.001
**Body image**		
Item 12	0.864	< 0.001
Item 11	0.883	< 0.001
Item 10	0.801	< 0.001
Item 9	0.785	< 0.001
**Arm symptom scale**		
Item 19	0.704	< 0.001
Item 18	0.487	< 0.001
Item 17	0.715	< 0.001
**Breast symptom**		
Item 21	0.726	< 0.001
Item 20	0.785	< 0.001
Item 23	0.567	< 0.001
Item 22	0.770	
**Sexual functioning**		
Item 14	0.677	< 0.001
Item 15	0.904	< 0.001
**Upset by hair loss**		
Item 5	0.707	< 0.001
**Future perspective**		
Item 13	0.893	< 0.001

## Discussion

This study showed that the QLQ-BR45 is a reliable and valid tool to assess the QoL of breast cancer patients. Previous studies have been conducted on the validity and reliability of the Amharic version of the QLQ-BR23. However, since the development of the QLQ-BR23 questionnaire, there have been major advances in the diagnosis and treatment of breast cancer, requiring the update of QLQ-BR23 to QLQ-BR45. The latter includes an additional 22 items, which were added to the original version.

The translation of the QLQ-BR45 was performed in collaboration with the EORTC translation team, and it followed the translation procedure developed by the EORTC QLG^17^. As a result of the translation process, the final version was linguistically and conceptually comprehensible to people of all education levels, culturally acceptable, and reflected the wording and structure of the original English version, as well as the standard layout and formatting of EORTC questionnaires. The reviewers of the EORTC quality of life translation team approved the Amharic translation and the procedure employed.

The average time required to complete the translated version of the questionnaire was 8 min (SD 2.8 min), which is comparable to the result of a previous study (9.2 min, SD 4.7 min)^7^. Both of these tools were developed by the EORTC and follow the same questionnaire development guidelines, and the number of items is almost the same.

In this study, the overall reliability of the questionnaire was 0.80, and all of the domains had an acceptable internal consistency value greater than 0.7, which is consistent with the phase III international update of QLQ-BR23^9^. This indicates that the internal consistency of the item is acceptable^23,28^. However, the arm symptom scale had a low internal consistency coefficient, similar to that observed for Moroccan breast cancer patients^29^. This can be explained by the inclusion of different areas of the body in the scale, such as the arm, shoulder, and hand. The overall test–retest reliability of all domains was 0.768, which was not satisfactory. One possible explanation is that the newly diagnosed breast cancer patients did not have much knowledge of the disease on their initial admission, but they gained more knowledge about the severity of the disease and its poor prognosis in the following days. Moreover, patients may have started taking symptom relief medication by the second assessment, such as pain relief medication. In addition, having an inconsistent environment for participants, such as being in a hurry during the test and mood instability, might have impacted the subjective assessment of QoL.

Multitrait scaling analysis showed that almost all of the items had stronger correlations with their own subscales than other subscales. This indicates strong convergent validity of the instruments. The magnitude of the correlation coefficients among all subscales was high (r = 0.40–1.00). However, item number 38 on the endocrine therapy scale had a low correlation (r < 0.4). The magnitude of discriminate validity was 88.7%. The most obvious scaling failure was observed for the systematic therapy side effect. This might have occurred because systematic therapy side effects are often nonspecific. This is in contrast to a previous study performed on breast cancer patients^10^.

In this study, the I-CVI score ranged from 0.83 to 1, and the S-CVI was found to be 0.94. An I-CVI score of 0.78 or higher is considered excellent^30^ and a S-CVI/Ave value of 0.90 or higher is acceptable^20^. Therefore, all items were considered relevant and had an excellent content validity score.

The interscale correlation value showed that the newly added QLQ-BR45 scales were not strongly correlated with the existing scales of the QLQ-BR23, similar to a previous update study on QLQ-BR23^9^. In this study, the endocrine therapy and systematic therapy side effect scales were strongly correlated with most of the other subscales, and strong correlation coefficients (r = 0.735) were found between the sexual enjoyment and sexual functioning scales. This strong correlation might result from both scales dealing with issues related to sexuality. Most other scales were correlated moderately or weakly with each other. The moderate or weak correlation of scales indicates that there are distinct components of the BR45 construct.

The external convergent validity correlation coefficient between EORTC QLQ-BR45 and EORTC QLQ-C30 was under 0.70. The systematic therapy side effect scale was correlated with the QLQ-C30 fatigue, nausea and vomiting, and pain scales. This correlation might be because both are symptom scales. The breast symptom and the arm symptom scales also had strong correlations with the pain scale of the QLQ-C30. A similar study performed in Turkey showed strong correlations between the symptom scales of QLQ-BR23 and QLQ-C30^10,31^. This might be explained by the two dimensions being conceptually related. Conversely, the symptom scales of EORTC QLQ-BR45 were more strongly correlated with the corresponding scales of EORTC QLQ-C30 than the functional scales^10^. Furthermore, correlations involving the sexual functioning, sexual enjoyment and endocrine sexual scales were low (r < 0.40). In Ethiopia, sexual-related issues are a sensitive topic, and patients often do not want to respond to related items. In the current study, 80% of the hypothesised scale structures between QLQ-BR45 and QLQ-C30 were not significantly correlated. However, the moderate and low correlation coefficients in the other domains of the QLQ-BR45 and QLQ-C30 suggest that the subscales were assessing distinct components of the QoL construct.

Known-group validity was examined for different groups, such as age, residential status, stage of disease, and treatment modalities. As expected, the mean functioning scales (sexual functioning and sexual enjoyment) had significantly lower scores in patients with metastatic disease compared to those in an early stage of disease, indicating that the QLQ-BR45 Amharic version is able to differentiate between patients with various disease severities. This is in line with a study conducted in Korean breast cancer patients^32^. Study participants aged over 45 years reported better sexual functioning, sexual enjoyment, body image, and future perspectives and a lower symptom scale (upset by hair loss) than participants younger than 45 years. Conversely, a study conducted in China showed that participants aged older than 50 years reported worse physical, role, and cognitive functioning and more sleep-related symptoms than those younger than 50 years^33^. This might be explained by differences in the tools used by the studies. Endocrine sexual-related function was lower in participants from rural places than urban places. This might be due to a lack of access to healthcare services and late detection of cancer.

Confirmatory factor analysis is a statistical technique used to test the hypothesised scale structure that assesses whether a relationship exists between the observed variables and their underlying latent constructs^34^. The hypothesised scale structure verified by CFA includes the 22 new items proposed by the EORTC QLQ study group, which showed no strong correlations with the existing scales of the QLQ-BR23^9^. It is recommended that a combination of measures should be used to test the hypothesised scale structure, such as the RMSEA, CFI, SRMER, and CMIN^35^. A RMSEA value less than 0.06 indicates an “excellent fit”, a value between 0.06 and 0.08 indicates an ‘acceptable fit’, and a value greater than 0.08 indicates an “unacceptable fit”. CFI and TLI values close to 0.95 and SRMR lower than 0.08 reflect a good fit of the model to the data^36^. Thus, based on these criteria, the constructs of the instrument demonstrated an excellent fit to the model.

Therefore, the results of multitrait scaling analysis and confirmatory factor analysis confirmed the hypothesised scale structure, indicating that the Amharic translation of the items and their response choices are appropriate and that scale scores could contribute to cross-cultural comparisons.

### Strengths and limitations of the study

Globally, this is the first validation study done on the newly updated QLQ-BR45. Forward and back translation of the new tool was carried out according to the EORTC translation guidelines. The study used different reliability and validity tests, including internal consistency reliability, test–retest reliability, and content, convergent, divergent, concurrent, and clinical validity, to test the reliability and validity of the tool. Furthermore, an adequate sample size was used, which is an improvement on previous validation studies performed in Ethiopia, in which the sample size was calculated according to rule of thumb. Robust AMOS software was used to conduct the confirmatory factor analysis.

Despite its strengths, this study also has some limitations. The QLQ-BR45 is a newly updated tool from the EORTC, and no other validation studies have been conducted, which makes it difficult to compare the results of this study to other studies. We have tried to overcome this limitation by comparing the results of this study to other related studies on previous versions of the questionnaire (QLQ-BR23 and QLQ-C30) conducted in Ethiopia and other countries. The responsiveness over time was not assessed in the current study. Furthermore, the test–retest reliability result was not satisfactory. We recommend that additional studies should be carried out with patients under active treatment to document the responsiveness and perform test–retest assessments at other time points. A phase IV international field study is currently being conducted on the QLQ-BR45. Therefore, additional studies may be needed if the tool is modified after the phase IV study is complete.

## Conclusion

The Amharic version of the EORTC QLQ-BR45 was found to be reliable, acceptable, and valid for assessing the QoL of breast cancer patients in Ethiopia. However, further studies on the responsiveness of the EORTC QLQ-BR45 and performing test–retest analysis while the patient is in a consistent environment with a stable mood are recommended.

## Data Availability

All data generated or analyzed during this study are included in this published article.

## Abbreviations

CFI: Comparative fit index
CVR: Content validity ratio
EORTC: European Organization for Research and Treatment of Cancer
ICC: Intraclass correlation
I-CVI: Item-level content validity index
QLQ: Quality of life questionnaire
QoL: Quality of life
RMSEA: Root mean square error of approximation
SRMR: Standardised root mean square residual
S-CVI: Scale-level content validity index
SD: Standard deviation
WHO: World Health Organization
