# Author Correction: Translation and validation of the EORTC QLQ-BR45 among Ethiopian breast cancer patients

**DOI:** 10.1038/s41598-022-25896-7

**Published:** 2022-12-16

**Authors:** Mikiyas Amare Getu, Panpan Wang, Eva Johanna Kantelhardt, Edom Seife, Changying Chen, Adamu Addissie

**Affiliations:** 1 The First Affiliated Hospital, Zhengzhou University, Zhengzhou, 450052 Henan China; 2 School of Nursing and Health, Zhengzhou University, Zhengzhou, 450001 Henan China; 3 Institute of Medical Epidemiology, Biostatistics and Informatics, Martin-Luther-University, Halle (Saale), Germany; 4 Department of Gynecology, Martin-Luther-University, Halle (Saale), Germany; 5 Department of Oncology, Tikur Anbessa Specialized Hospital, College of Health Science, Addis Ababa University, Addis Ababa, Ethiopia; 6 Department of Preventive Medicine, School of Public Health, College of Health Sciences, Addis Ababa University, Addis Ababa, Ethiopia

Correction to: *Scientific Reports* https://doi.org/10.1038/s41598-021-02511-9, published online 29 July 2022

The original version of this Article contained errors in Table 1.

The values for “Above secondary school education” in section “Educational status” in column “Frequency” were incorrectly given as “56” instead of “57”, and in column “Percentage” the values were incorrectly given as “23.4” instead of “23.8”. Moreover, in column “Percentage”, the value for “No education” was rounded down from “31.4” to “31.2”, and the value for “Primary school education” was rounded down from “21.3” to “21.2”. The correct and incorrect values appear below.

Additionally, the data for “Protestant” in section “Religion” was omitted. The value “50” was added in column “Frequency” and the value “20.8” was added in column “Percentage”. Moreover, in column “Percentage”, the value for “Other^†^” was rounded down from “1.3” to “1.2”. The correct and incorrect values appear below.

Incorrect:

**Table:** 

Variable	Frequency	Percentage
**Educational status**		
No education	75	31.4
Primary school education	51	21.3
Secondary school education	57	23.8
Above secondary school education	56	23.4
**Religion**		
Orthodox Christianity	142	59.2
Muslim	41	17.1
Catholic	4	1.7
Other^†^	3	1.3

Correct:

**Table:** 

Variable	Frequency	Percentage
**Educational status**		
No education	75	31.2
Primary school education	51	21.2
Secondary school education	57	23.8
Above secondary school education	57	23.8
**Religion**		
Orthodox Christianity	142	59.2
Protestant	50	20.8
Muslim	41	17.1
Catholic	4	1.7
Other^†^	3	1.2

The original Article has been corrected.

